# The *Pseudomonas* Quinolone Signal Inhibits Biofilm Development of *Streptococcus mutans*

**DOI:** 10.1264/jsme2.ME14140

**Published:** 2015-04-09

**Authors:** Tomohiro Inaba, Hiromu Oura, Kana Morinaga, Masanori Toyofuku, Nobuhiko Nomura

**Affiliations:** 1 Graduate School of Life and Environmental Sciences, University of Tsukuba1-1-1 Tennodai, Tsukuba, Ibaraki 305–8572Japan

**Keywords:** biofilm, quorum sensing, *Streptococcus mutans*

## Abstract

Bacteria often thrive in natural environments through a sessile mode of growth, known as the biofilm. Biofilms are well-structured communities and their formation is tightly regulated. However, the mechanisms by which interspecies interactions alter the formation of biofilms have not yet been elucidated in detail. We herein demonstrated that a quorum-sensing signal in *Pseudomonas aeruginosa* (the *Pseudomonas* quinolone signal; PQS) inhibited biofilm formation by *Streptococcus mutans*. Although the PQS did not affect cell growth, biofilm formation was markedly inhibited. Our results revealed a unique role for this multifunctional PQS and also indicated its application in the development of prophylactic agents against caries-causing *S. mutans*.

A biofilm is an aggregate of cells that is attached to a surface and enclosed in an extracellular matrix. One of the major problems associated with biofilms is that they can cause chronic infections. A previous study estimated that 65% of all bacterial infections involve biofilms (11). It has also been estimated that more than 500 bacterial species exist in the human oral cavity (4, 10). These diverse bacteria have been suggested to communicate with each other in the oral cavity and establish an oral multispecies biofilm (5). Bacterial signaling molecules were recently shown to have several effects on other bacterial species, including antibiotic activity and an influence on membrane vesicle production (12, 18, 19). Therefore, complex interactions may occur between oral bacteria and other bacteria through signaling molecules. *Pseudomonas aeruginosa* has been identified as one of the indigenous bacteria in the oral cavity (16). Furthermore, the signaling molecule of *P. aeruginosa*, the *Pseudomonas* quinolone signal (PQS), was previously shown to affect other bacterial species (14, 18, 19). However, the effects of the PQS on biofilm formation by other bacterial species have not yet been clarified. By examining the interaction between *P. aeruginosa* and *Streptococcus mutans*, we herein demonstrated that the PQS inhibited biofilm formation by *S. mutans* (Fig. 1A). The growth of *S. mutans* in liquid and solid media was not affected by the addition of the PQS (Fig. 1B–D). These results suggested that biofilm formation by *S. mutans* was inhibited by the PQS because the ability of the bacteria to attach to the surface was reduced (Fig. 1B). Microscopic observations also revealed that autoagglutination was not induced by the addition of the PQS (data not shown). Therefore, we tested the effects of the PQS on the formation of biofilms on hydroxyapatite (HA) disk surfaces. Hydroxyapatite is a major component of human teeth and is a useful substratum that experimentally imitates natural oral conditions for biofilm formation assays. The exact nature of the interaction between biofilms and HA needs to be clarified in order to obtain a better understanding of biofilm formation in natural settings. We previously reported that this new approach for observing biofilms allowed for the simultaneous visualization of biofilms and its attached substratum (3, 21). In the present study, we also demonstrated the usability of our observation technique for biofilm-measuring assays. Using the biofilm formation assay, we showed that the formation of biofilms by *S. mutans* on HA disks with the addition of the PQS was markedly less than that on glass surfaces (Fig. 2A and B). In *S. mutans*, gene expression levels have been shown to differ in a manner that is dependent on the type of attached surface (17). This finding suggests that the PQS may affect specific genes that play important roles in biofilm formation on HA disks. Moreover, the effects of the PQS on other bacteria were previously attributed to the iron-chelating activity of PQS molecules (8). Therefore, the influence of the chelating activity of the PQS was examined by the addition of iron (III) chloride. The addition of iron (III) chloride did not affect the biofilm inhibitory effects of the PQS (Fig. 1A, 2A and 2B). These results indicated that the inhibition of biofilm formation by the PQS was not caused by its iron-chelating activity; therefore, generation of the PQS by *P. aeruginosa* may be favorable for survival in the oral cavity. *S. mutans* uses extracellular polysaccharides to adhere to human teeth (15). Some compounds are known as biofilm inhibitors because of their inhibitory effects on the glycosyltransferase enzymes essential for the synthesis of polysaccharides (1, 6, 7, 9). However, the results of our Congo red assay revealed that the PQS did not affect the amount of extracellular polysaccharide produced, suggesting that activities associated with the synthesis of polysaccharides, such as glucosyltransferase activity, were not inhibited by the PQS (Fig. S1). We then attempted to determine which stages of biofilm formation were inhibited by adding the PQS at 0, 1, 2, 4, and 6 h after inoculation. The inhibition of biofilm formation by the PQS was only observed in the initial stage of biofilm formation (from 0 to 1 h after inoculation) (Fig. 2C). However, a microscopic visualization revealed that the attached cell biomass (1.5 h after inoculation) was not disrupted by the addition of the PQS (data not shown). These results suggested that the PQS inhibited the initial stage of biofilm development, but not initial attachment. In the present study, we showed that PQS inhibited biofilm formation by *S. mutans* and that it also inhibited biofilm formation without inhibiting cellular growth. These results implied that the development of a biofilm-specific inhibitor without the emergence of resistant strains was possible. Although the PQS is considered to be a quorum-sensing (QS) signal, recent findings have indicated that PQS is multifunctional (14, 18, 19). Our study adds further information for the unique effects of this signal on *S. mutans* biofilm formation and will contribute to a better understanding of interspecies interactions through chemical signals. *S. mutans* is known to cause serious infectious diseases, such as infective endocarditis and aspiration pneumonia (13, 20). Therefore, preventing the formation of biofilms by *S. mutans* is very important for systemic health care. A deeper understanding of the biofilm inhibition mechanism employed by the PQS may contribute to the development of new agents to control biofilm formation by *S. mutans*.

## Supplementary Information



## Figures and Tables

**Fig. 1:**
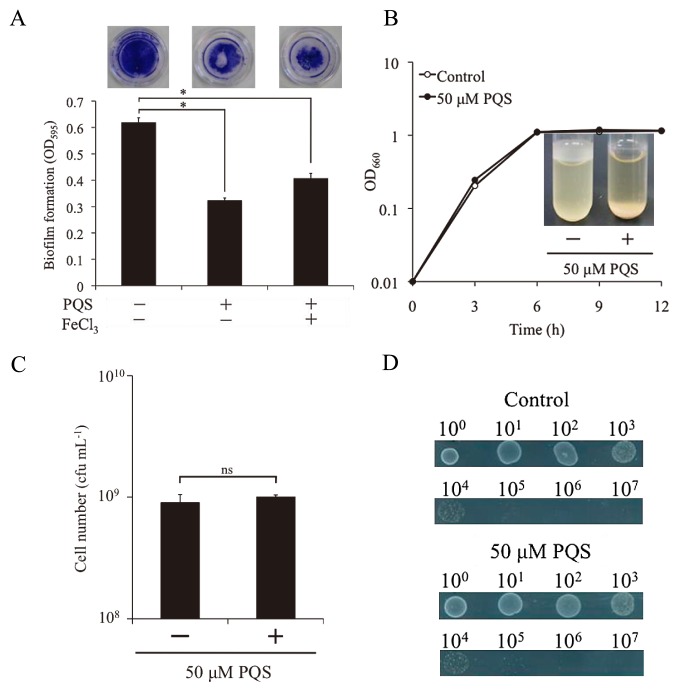
Effects of PQS on biofilm formation, growth, and cellular viability in *S. mutans* NCIMB702062. (A) Effects of PQS on biofilm formation on glass-based dishes in the presence or absence of iron. Biofilms were stained with crystal violet and quantified by measuring OD_595_. Biofilms were incubated in tryptic soy broth (TSB medium (Becton Dickinson, Sparks, MD, USA) at 37°C for 12 h. PQS and FeCl_3_ were added to the cultures at final concentrations of 50 μM, respectively. *P<0.01 (*t*-test) (B) Growth curve of *S. mutans* with or without 50 μM PQS. Each tube was incubated in TSB medium at 37°C in a static culture. Pictures of tubes were taken at 12 h. (C) Viable cell counts after a 12-h incubation in liquid TSB medium at 37°C in a static culture. After being incubated, cells were harvested and diluted with saline. A cell suspension was plated on TSB agar and cultured at 37°C for 24 h. Three independent experiments were carried out, and data represented are means ± standard deviations of triplicate assays. ns, the difference was not significant. (D) Growth of *S. mutans* on TSB medium (1.5% agar) with or without 50 μM PQS. Cells were incubated at 37°C for 24 h. The data shown are representative of three independent experiments.

**Fig. 2:**
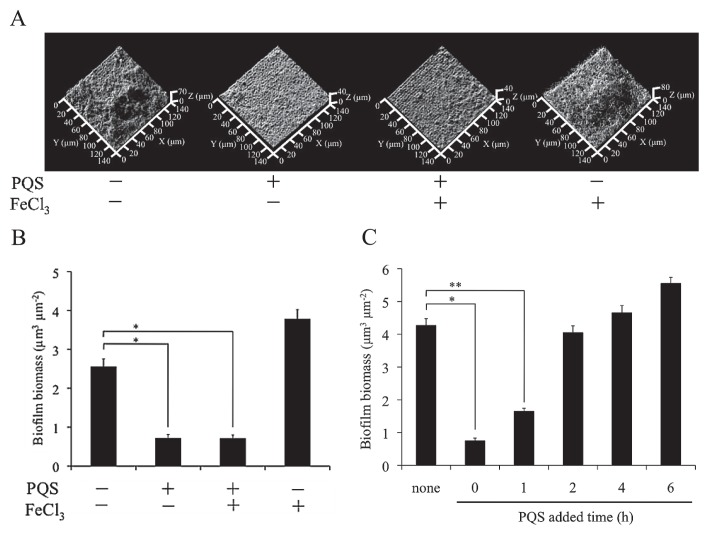
Effects of the PQS on biofilm formation. (A) Observation of the biofilm structure on HA surfaces. The visualization was performed by confocal reflection microscopy. At least five pictures were taken per sample and representative pictures were shown. (B) Quantification of the microscopic image shown in (A). Quantification was performed using the COMSTAT program (2). *P<0.01 (*t*-test) (C) Effects of the time-of-addition of the PQS on the inhibition of biofilm formation by *S. mutans*. The PQS was added at the time indicated on the X-axis, and the biofilm was then allowed to form for a total of 12 h. Biofilms were incubated in TSB medium at 37°C for 12 h. The PQS was added to the cultures at a final concentration of 50 μM, and, in panels A and B, FeCl_3_ was added at a final concentration of 150 μM. respectively. The averages are based on three or more independent determinations, and the standard errors are indicated. *P<0.01, **P<0.05 (*t*-test). The data shown are representative of three independent experiments.
