# Supplementary material for: The Pseudomonas Quinolone Signal Inhibits Biofilm Development of Streptococcus mutans
Source: Microbes Environ. 2015 Apr 9;30(2):189–91. doi: 10.1264/jsme2.ME14140 (PMC4462930; doi:10.1264/jsme2.ME14140)
Supplement: Supplementary file 1 [file 30_189_s1.pdf]

Supplemental Fig. 1. Influence of the PQS on EPS production. The amount of Congo red adsorbed by cells. Plates were incubated for 12 h at 37°C and 12 h at room temperature. Cells that adsorbed Congo red were extracted using ethanol, and the amount of Congo red was detected by an adsorption spectrometer. The data represented are means ± standard deviations of triplicate assays. ns, the difference was not significant.

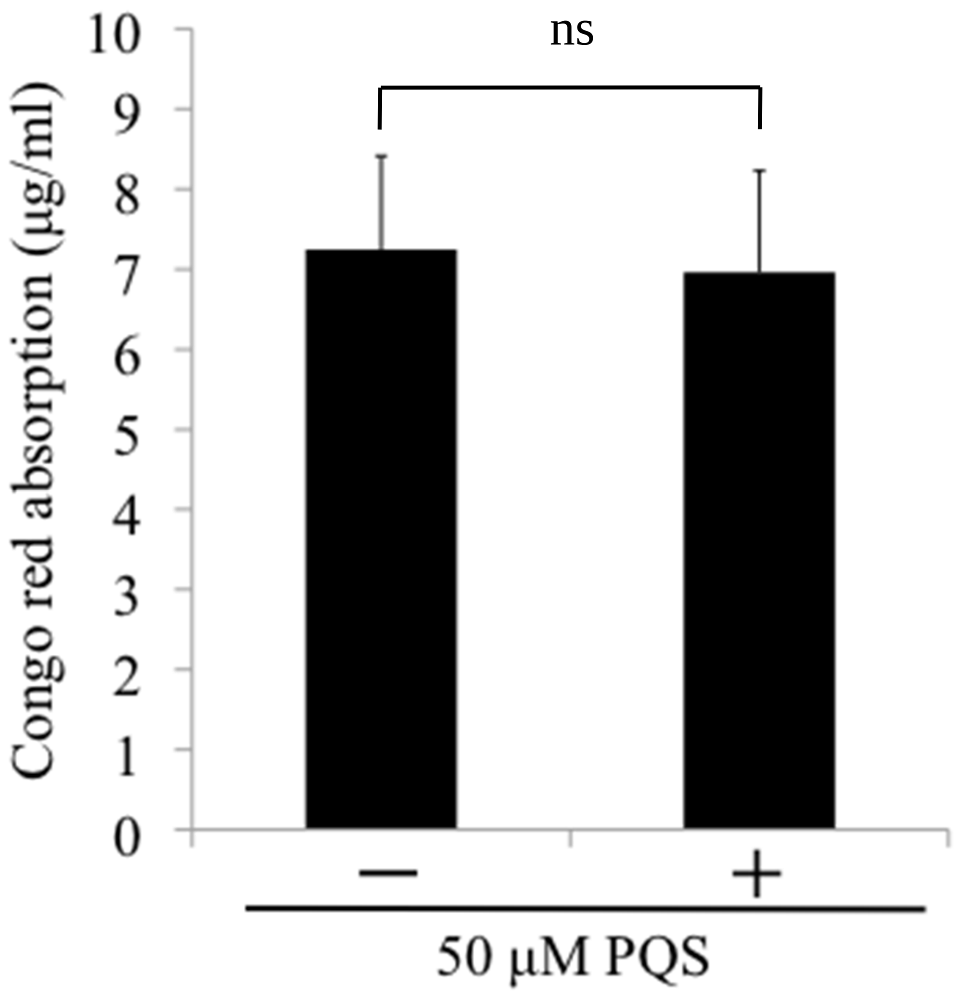
ns
Congo red absorption (μg/ml)
0
1
2
3
4
5
6
7
8
9
10
−
+
50 μM PQS
